# Multi-omics insights into immune tolerance at the maternal–fetal interface in recurrent pregnancy loss: mechanisms, integration challenges, and translational perspectives

**DOI:** 10.3389/fimmu.2026.1811970

**Published:** 2026-04-29

**Authors:** Mengqiu Shao, Yiting Zhang, Haixia Tang, Ze Zhou, Manyin Zhai, Xiaoyu Zhou, Xiaoyu Bi, Jiabao Liao, Caiyan Zhang, Lijuan Jiang

**Affiliations:** 1 Yunnan University of Chinese Medicine, Kunming, China; 2 Jiaxing Hospital of Traditional Chinese Medicine, Jiaxing, China; 3 The First Affiliated Hospital of Yunnan University of Chinese Medicine, Kunming, China

**Keywords:** biomarker discovery, decidual immune microenvironment, immune dysregulation, maternal–fetal immune tolerance, multi-omics, recurrent pregnancy loss, spatial immunology

## Abstract

Recurrent pregnancy loss (RPL) is a heterogeneous reproductive disorder in which dysregulation of maternal–fetal immune tolerance, aberrant decidual immune remodeling, and altered inflammasome signaling have been implicated within a complex multi-omics landscape. Multi-omics profiling (genomics, epigenomics, single-cell/spatial transcriptomics, proteomics, metabolomics, microbiome analyses, and immunomics) is increasingly being used to characterize mechanistic heterogeneity in RPL and to support biomarker discovery and immune-informed stratification. Genomic studies have associated chromosomal abnormalities and pathogenic variants with early embryonic developmental failure, while epigenomic profiling has highlighted aberrant methylation patterns and imprinting disturbances. Single-cell and spatial transcriptomics have revealed altered cellular composition and disrupted communication among decidual stromal cells, uterine natural killer (uNK) cells, macrophages, regulatory T cells (Treg), T helper 17 (Th17) cells, and trophoblast lineages. Proteomic and metabolomic studies have further identified immune–metabolic signatures associated with impaired trophoblast function and vascular remodeling, while emerging microbiome studies suggest a gut–reproductive axis that may modulate systemic immune homeostasis. Integration of multi-omics datasets with computational frameworks (e.g., weighted gene co-expression network analysis (WGCNA), multi-omics factor analysis (MOFA), and deep-learning models may improve RPL subtype classification, risk prediction, and the identification of potentially actionable pathways. However, current studies remain limited by small cohort sizes, especially in single-cell datasets, cross-platform heterogeneity, insufficient longitudinal validation, and a lack of multicenter reproducibility. Future work should prioritize standardized multi-omics pipelines, clearer evidence stratification, and immune-centric analytical frameworks to improve the robustness and translational relevance of RPL research. These advances may ultimately support immune-informed risk assessment and contribute to the gradual development of more individualized management strategies for RPL.

## Graphical Abstract

**Figure:**
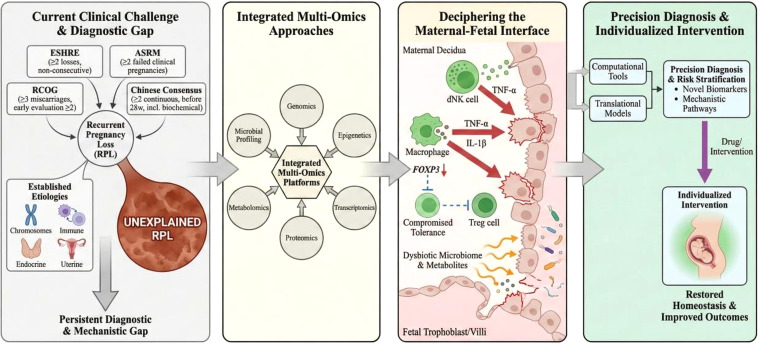

## 1 Introduction

Recurrent pregnancy loss (RPL) is a complex reproductive disorder affecting approximately 1% to 5% of women of reproductive age (1). Diagnostic definitions vary across professional guidelines. The European Society of Human Reproduction and Embryology (ESHRE) defines RPL as ≥2 pregnancy losses, without requiring continuity or the same partner (2). The American Society for Reproductive Medicine (ASRM) defines it as ≥2 clinically confirmed pregnancy failures via ultrasound or pathology, also without requiring continuity (3). The Royal College of Obstetricians and Gynaecologists (RCOG) defines it as ≥3 miscarriages in the first trimester (≤13 weeks), although evaluation can be considered after ≥2 miscarriages (4). The Chinese (2022) expert consensus defines it as ≥2 consecutive pregnancy losses with the same spouse before 28 weeks of gestation, including biochemical pregnancies (5).

The etiology of RPL is multifactorial and highly complex. Beyond well-defined factors such as chromosomal abnormalities, maternal immune dysregulation, endocrine disorders, and uterine anatomical abnormalities (6), a considerable proportion of cases remains classified as “unexplained” (7), highlighting substantial biological heterogeneity and unresolved mechanistic complexity.

Traditional single-omics approaches are often insufficient to capture the multi-level molecular and cellular networks involved in RPL, thereby limiting mechanistic interpretation and clinically meaningful stratification (8). Therefore, there is an increasing need to integrate multi-omics and systems biology strategies to identify convergent pathways, refine biologically meaningful classifications, and inform future hypothesis-driven and translational research.

**Figure 1:**
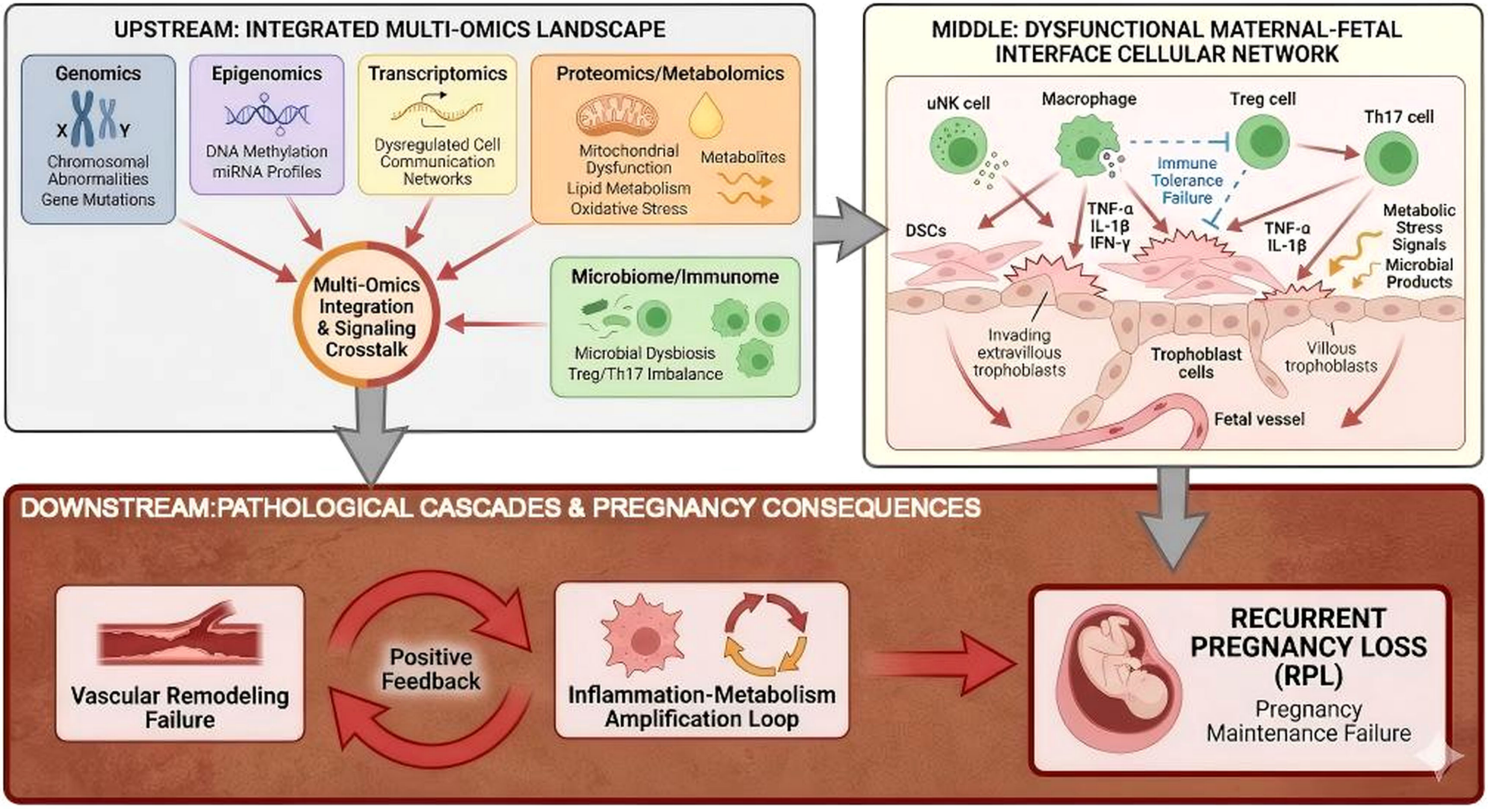
Integrated multi-omics landscape of immune tolerance disruption at the maternal–fetal interface in recurrent pregnancy loss (RPL). This schematic summarizes how multiple omics layers—including genomics, epigenomics, transcriptomics, proteomics/metabolomics, and microbiome/immunome profiling—can be integrated to characterize dysregulated cellular communication and immune signaling at the maternal–fetal interface. The upstream panel highlights representative molecular domains implicated in RPL, such as chromosomal abnormalities, DNA methylation changes, altered cell–cell communication networks, mitochondrial dysfunction, metabolic imbalance, and microbial dysbiosis. The middle panel illustrates a dysfunctional decidual cellular network involving decidual stromal cells (DSCs), uterine natural killer (uNK) cells, macrophages, regulatory T cells (Treg), T helper 17 (Th17) cells, and trophoblast populations. The downstream panel depicts how these perturbations may converge on vascular remodeling failure, inflammation–metabolism amplification, and pregnancy maintenance failure. This figure provides a conceptual synthesis of current multi-omics findings and is intended to illustrate an integrative disease framework rather than a definitive causal pathway. DSCs, decidual stromal cells; IFN-γ, interferon-gamma; IL-1β, interleukin-1 beta; TNF-α, tumor necrosis factor-alpha; Treg, regulatory T cell; Th17, T helper 17 cell; uNK, uterine natural killer cell.

This review systematically summarizes recent advances in the application of multi-omics technologies to the study of RPL, with a particular focus on immune–metabolic–microbiome interactionsat the maternal–fetal interface. It highlights representative omics-derived signals relevant to risk assessment and mechanistic stratification, and evaluates the emerging potential of AI-assisted analysis, multi-omics modeling, and translational platforms in biomarker discovery and immune-informed management of RPL, thereby providing a conceptual framework and future research directions for mechanism-oriented integration (**Figure 1**).

Therefore, this review integrates multi-omics and spatial immunology perspectives to provide an updated framework for understanding RPL as a complex immune-associated reproductive disorder and to highlight immune-centric directions for future stratification and intervention research.

## 2 Literature search strategy

This review was based on a structured literature search of studies related to RPL, maternal–fetal immune tolerance, and multi-omics applications in reproductive immunology. The search was conducted primarily in PubMed, Web of Science, and Embase, and covered literature published up to January 2026.

The core search terms included combinations of the following keywords: “recurrent pregnancy loss,” “recurrent miscarriage,” “maternal–fetal interface,” “immune tolerance,” “decidual immune cells,” “uterine natural killer cells,” “regulatory T cells,” “Th17 cells,” “inflammasome,” “multi-omics,” “genomics,” “epigenomics,” “single-cell transcriptomics,” “spatial transcriptomics,” “proteomics,” “metabolomics,” “microbiome,” and “immunomics.” Additional manual screening of reference lists from relevant original articles and review papers was also performed to identify representative studies of high relevance.

Priority was given to peer-reviewed English-language studies with clear relevance to maternal–fetal immune regulation and the mechanistic interpretation of RPL. Where available, preference was given to human studies, including cohort studies, omics-based profiling studies, and translational investigations. Representative animal studies and *in vitro* mechanistic studies were also included when human evidence was limited but the findings were relevant for understanding immune-regulatory mechanisms. Studies with limited relevance to immune tolerance at the maternal–fetal interface, insufficient methodological information, or predominantly descriptive findings without mechanistic significance were not prioritized.

Given the rapid development of this field, emphasis was placed on recent and methodologically informative studies, particularly those contributing to the understanding of immune-cell interaction networks, immune–metabolic and microbiome-associated regulation, inflammasome-related pathways, and multi-omics integration strategies in RPL. This review therefore aims to provide a structured and critical synthesis of current evidence rather than an exhaustive systematic review.

## 3 Current status of the integrated application of multi-omics in RPL

In recent years, with the development of high-throughput omics platforms and systems biology frameworks, RPL research has gradually shifted from single-mechanism and single-target approaches toward a multi-layer, systems-level integrative paradigm (9). This shift has encouraged the application of multi-omics strategies to integrate information derived from multiple biological layers, including genomic variations, epigenomic modifications, transcriptomic profiles, proteomic regulation, metabolic signatures, immune responses, and microbiome profiles. Integrating multiple omics layers may help identify key molecular nodes and signaling pathways associated with RPL, thereby providing a conceptual and methodological basis for mechanistic stratification, biomarker discovery, and future individualized management strategies.

### 3.1 Genomics and epigenomics

Chromosomal structural abnormalities in embryos and parents are among the earliest recognized pathogenic factors in RPL (10). Balanced translocations or inversions can lead to abnormal gametes, resulting in aneuploidy or chromosomally unbalanced embryos, and have been reported in a subset of RPL cases, although the estimated prevalence varies across studies (11). Susceptibility gene variants such as factor V Leiden (FVL) and methylenetetrahydrofolate reductase (MTHFR) have been associated with thrombosis and placental perfusion defects (12), suggesting that genetic factors may also contribute to the maintenance of homeostasis at the maternal–fetal interface.

Collectively, genomic alterations may shape the immune landscape at the maternal–fetal interface by influencing antigen presentation, immune tolerance pathways, and trophoblast–immune cell interactions. These findings suggest that genetic susceptibility in RPL is closely intertwined with immune regulatory networks rather than acting solely through embryonic defects.

Epigenomic studies have further emphasized the importance of regulatory disruption at the noncoding level. In embryos, an imbalance characterized by hypomethylation of the imprinted maternally expressed transcript(H19) and hypermethylation of insulin-like growth factor 2 (IGF2) has been reported to disrupt trophoblast cell cycle progression, invasion, and differentiation (13). From a paternal perspective, a high sperm DNA fragmentation index has been associated with impaired embryo development (14). From a maternal perspective, elevated levels of microRNAs such as miR-517a/b have been detected in the uterine fluid of patients with RPL. These microRNAs may target and inhibit cyclooxygenase-2 (COX-2) and downstream decidualization pathways, thereby contributing to endometrial insufficiency (13).

Epigenetic dysregulation may therefore represent an important interface between environmental stressors and immune imbalance by modulating cytokine signaling, regulatory T-cell stability, and decidual immune adaptation. Understanding these epigenetic–immune interactions may help explain the heterogeneity of immune phenotypes observed in RPL.

However, current genomic and epigenomic findings remain limited by cohort heterogeneity, inconsistent phenotyping, and insufficient functional validation in human decidual tissues.

### 3.2 Transcriptomics and single-cell omics

A central feature of RPL pathophysiology is disturbance of the interaction network at the maternal–fetal interface. Although traditional bulk RNA sequencing can reveal differences in gene expression at the tissue level, it cannot resolve cellular heterogeneity or spatially localized expression patterns within the local microenvironment. Transcriptome analyses of decidual tissue indicate that maternal–fetal interactions are altered in RPL, particularly through dysregulation of gene modules related to immune responses, complement pathways, and intercellular communication (15–17). Single-cell RNA sequencing studies have further revealed imbalanced cell–cell interactions at the maternal–fetal interface, particularly between decidual stromal cells (DSCs) and uterine natural killer (uNK) cells in RPL. When TGF-β signaling is reduced, DSCs may fail to direct uNK cells toward a tolerant state. In addition, reduced secretion of β-galactoside-binding lectin 9 (Galectin-9) from uNK cells may weaken their supportive effects on extravillous and villous trophoblast cells, thereby compromising the structural and regulatory integrity of the maternal–fetal interface (18). In trophoblast subgroups, an imbalance between invasive and secretory trophoblasts has been associated with reduced expression of matrix metalloproteinase-9 (MMP-9), which may contribute to defective remodeling of the uterine spiral arteries (19). Attenuated vascular endothelial growth factor A (VEGFA) signaling may aggravate perfusion insufficiency and contribute to focal ischemia and impaired embryonic growth (20). These transcriptomic maps highlight key pathological links at the maternal–fetal interface in RPL and provide a basis for molecular classification based on expression profiles, which maysupport future risk stratification and individualized management.

Single-cell and spatial transcriptomic analyses consistently suggest that immune-cell crosstalk, rather than isolated cellular dysfunction, may play a major role in disease progression. These data support the concept that disrupted communication among decidual immune populations is closely associated with the breakdown of maternal–fetal immune tolerance.

Nevertheless, current single-cell and spatial transcriptomic studies in RPL remain constrained by small sample sizes, sampling-time variability, and cross-study differences in cell annotation and analytical pipelines.

### 3.3 Proteomics and metabolomics

Proteomic analyses have identified altered expression of functional proteins in tissues and body fluids from patients with RPL. Several pregnancy-associated proteins, including pregnancy-associated plasma protein A (PAPP-A), placental growth factor (PlGF), and annexin A5 (ANXA5), have been reported to bereduced in RPL cohorts, suggesting potential disruption of trophoblast function and immune-vascular regulation (21).The expression of plasminogen activator inhibitor-1 (PAI-1) and lipocalin-2 (LCN2), which exert procoagulant and proinflammatory effects, has been reported to be increased, potentially contributing to microthrombosis and inflammation at the maternal–fetal interface (22, 23). Metabolomic studies suggest that a subset of patients with RPL exhibit disturbances in glycolysis, the tricarboxylic acid (TCA) cycle, and lipid metabolism, in which downregulation of glucose transporter 1 (GLUT1) and glucose transporter 3 (GLUT3), together with an elevated lactate/pyruvate ratio, may reflect impaired glucose acquisition and utilization (24). In lipid metabolism, overexpression of sphingomyelinase (SMase) may increase ceramide production, which has been implicated in the activation of c-Jun N-terminal kinase (JNK) and p38 mitogen-activated protein kinase (p38 MAPK) signaling, ultimately contributing to trophoblast apoptosis (25). Moreover, disturbance in glycerophospholipid metabolism has been associated with impaired LPA–LPAR–PI3K/Akt signaling and reduced trophoblast invasiveness (26). Metabolic changes appear to represent an important component of RPL pathophysiology, and some reported marker panels have achieved an area under the curve (AUC) greater than 0.9 in independent exploratory datasets for non-invasive prediction of RPL (27).

Beyond biomarker discovery, proteomic and metabolomic signatures suggest coordinated immune–metabolic reprogramming at the maternal–fetal interface. Such immune–metabolic coupling may represent a mechanistic bridge linking vascular dysfunction, inflammation, and trophoblast stress in RPL.

However, proteomic and metabolomic studies remain sensitive to platform variability, pre-analytical differences, and limited multicenter validation, which may affect reproducibility across cohorts.

### 3.4 Microbiome and immunomics

In patients with RPL, the reproductive tract microbiota has often been reported to show reduced *Lactobacillus* abundance, overgrowth of *Gardnerella* and anaerobes, and increased microbial diversity (28). Under conditions of microbial dysbiosis, the local immune barrier may be impaired, with increased levels of pro-inflammatory factors such as interleukin-6 (IL-6) and tumor necrosis factor-α (TNF-α). This disruption may interfere with decidual–trophoblast interactions and has been associated with reduced implantation success (29). Gut microbiota may also regulate peripheral immune homeostasis via short-chain fatty acids (SCFAs) (30). In RPL, reduced abundance of butyrate-producing bacteria (e.g., *Bifidobacterium* and *Akkermansia*) may contribute to lower Treg proportions and relative expansion of Th17-associated responses, thereby compromising maternal–fetal immune tolerance (31). Immunomic analyses, including mass cytometry (CyTOF), generally indicate a shift toward a pro-inflammatory immune landscape in RPL, characterized by reduced regulatory T-cell representation and enhanced Th17-associated responses within the decidua. Although the magnitude of this imbalance varies across cohorts and analytical platforms, disruption of the Treg/Th17 axis is widely regarded as an important feature of impaired maternal–fetal immune tolerance (32, 33).The advent of spatial transcriptomics and high-dimensional flow cytometry has improved the identification of localized inflammation niches. Multiple inflammatory foci (<200μm) in RPL decidua have been reported to overlap with areas of placental dysfunction, suggesting that spatial immune profiling may become valuable for future RPL stratification and mechanistic assessment (34).

Emerging evidence further suggests that microbiome-derived signals may reshape systemic immune tone and influence decidual immune activation. The gut–reproductive axis may therefore represent a potential upstream regulator of immune tolerance failure in susceptible individuals.

Nevertheless, microbiome and immunomics studies remain sensitive to sampling location, contamination control, sequencing depth, and inter-cohort variation, which complicates reproducibility and interpretation.

### 3.5 Multi-omics integration and interaction

Omics integration is not merely the aggregation of layered datasets, but rather aims to identify cascade pathways and construct coordinated networks across biological levels. For instance, analyses of proteome–metabolome interactions have suggested that complement factor C3a (C3a) and lysophosphatidylcholine (LysoPC)may form a positive feedback loop and act synergistically in vascular endothelial injury (35). Methylation–transcription joint analyses have shown that hypermethylation of the forkhead box P3 (FOXP3) promoter region is closely associated with reduced FOXP3 expression in Treg cells, representing a characteristic signal of immune dysregulation in RPL (36). In terms of data analysis methods, multi-omics integration tools such as Multi-Omics Factor Analysis (MOFA), Weighted Gene Co-expression Network Analysis (WGCNA), and Data Integration Analysis for Biomarker Discovery using Latent Variable approaches (DIABLO) are widely used for feature extraction and module identification, thereby supporting mechanistic interpretation and subtype classification in complex diseases such as RPL (8).

Importantly, multi-omics integration suggests that immune dysregulation may emerge from coordinated alterations across genomic, epigenetic, transcriptomic, and metabolic layers rather than isolated pathway changes. Integrative analyses frequently converge on immune tolerance disruption, inflammasome activation, and immune–vascular crosstalk as major axes implicated in RPL. These findings reinforce the value of immune-centric systems biology frameworks for understanding disease heterogeneity and informing future immune-oriented stratification and therapeutic exploration.

However, multi-omics integration in RPL remains challenged by incomplete modality matching, differences in preprocessing pipelines, and limited availability of longitudinal datasets with paired clinical metadata.

### 3.6 Multi-omics diagnostic models and artificial intelligence–assisted analysis

Combining multi-omics data to construct prediction models is emerging as an important strategy for improving risk stratification in RPL. By integrating multidimensional information such as inflammatory and angiogenic factors, metabolite profiles, and reproductive tract microbiota, the inflammation–metabolism–microbiota axis may be reconstructed at the individual level. This reconstruction may enhance the ability to perform early screening and risk stratification (8, 37, 38). With the advancement of AI, methods such as random forest (RF), Extreme Gradient Boosting (XGBoost), and deep neural networks (DNNs) have been increasingly used in omics modeling. These methods may improve predictive performance, identify important variables, and enhance model interpretability (39).

Importantly, these AI-driven approaches may help reveal coordinated immune signaling patterns rather than isolated statistical predictors, highlighting the potential value of machine learning in exploring immune tolerance mechanisms underlying RPL.

Autoencoder-based deep embedding models are promising multi-omics integrators that have shown advantages in cross-modal data representation learning across multiple disease contexts and single-cell datasets (40). In RPL, decidual single-cell transcriptomes have been integrated with machine learning approaches to identify diagnostic-related molecular features and support patient stratification (41). In contrast, traditional machine learning models such as XGBoost may be more robust and interpretable under small- and medium-sized sample conditions and may be better suited for exploratory deployment in clinical high-throughput data settings.

Future research should focus on integrating interpretable AI into omics feature selection and clinical reasoning to address the “black box” limitation of deep models. In the future, an “omics + AI” platform could potentially be incorporated into RPL management workflows, forming a closed-loop process of “sampling–preprocessing–analysis–reporting–recommendation”. Such a platform would ideally connect to hospital electronic medical record (EMR) and assisted reproductive information systems, supporting automated data updates, dynamic scoring, and individualized decision support.

Although promising, the clinical implementation of AI-enabled multi-omics platforms faces major challenges, including cross-center data heterogeneity, variable sampling times, inconsistent preprocessing protocols, and privacy compliance. Establishing regulatory-compliant data-sharing mechanisms—such as those required by the General Data Protection Regulation (GDPR) and the Health Insurance Portability and Accountability Act (HIPAA)—together with developing multi-omics metadata standards and ethical interpretability frameworks, will be necessary before large-scale deployment becomes feasible.

Overall, multi-omics research offers an integrated framework for investigating RPL across genetics, metabolism, immunity, and microbiome-related domains. By constructing cross-level signaling maps and interaction networks, RPL research is gradually evolving from phenotypic description toward mechanistic interpretation and from risk identification toward more refined stratification. The next stage should focus on the clinical adaptation of spatial omics, real-time monitoring methods, and standardized multicenter databases to improve the translational robustness of future RPL diagnostic and management frameworks.

From an immunological perspective, AI-assisted multi-omics integration may not only improve predictive accuracy but also help reveal coordinated immune regulatory networks underlying RPL heterogeneity. Emerging models have increasingly highlighted immune tolerance disruption, inflammasome signaling, and immune–vascular interactions as important features associated with disease progression, thereby supporting further development of immune-informed stratification and exploratory immunomodulatory strategies.

At present, however, most AI-assisted multi-omics models in RPL remain exploratory, and their generalizability, prospective performance, and clinical utility require further validation in larger multicenter cohorts.

## 4 Immune signaling interactions and pathological network reconstruction at the maternal–fetal interface

Recent multi-omics and spatial immunology studies increasingly suggest that RPL may be better understood as a network-level immune disorder rather than a condition driven solely by isolated molecular defects. Importantly, these alterations appear to converge on immune signaling hubs that reshape maternal–fetal immune tolerance rather than acting as isolated molecular events.

Crosstalk among decidual immune cells, trophoblast populations, vascular endothelial pathways, and inflammasome-associated signaling may form a dynamic pathological network linking immune tolerance failure with vascular maladaptation and inflammatory amplification. Understanding these integrated immune signaling interactions provides a conceptual bridge between omics-level observations and future immune-informed therapeutic exploration.

During the establishment and maintenance of pregnancy, the maternal-fetal interface is a critical region influencing embryonic implantation, placental development, and pregnancy maintenance. It is primarily composed of DSCs, uNK cells, macrophages—including classically activated (M1) and alternatively activated (M2) subsets—Treg cells, and multiple trophoblast populations, including syncytial, invasive, and columnar phenotypes (42). In normal pregnancy, this interface coordinates multiple biological processes —including immune tolerance formation, vascular remodeling, and tissue repair adaptation—to maintain dynamic homeostasis during embryonic and placental development. RPL may reflect systemic disruption of this coordinated network at the maternal–fetal interface, with both structural and functional integrity being affected. It may be viewed as a major pathological endpoint at which aberrant signaling across multiple omics layers—including genetics, epigenetics, transcription, proteomics, metabolomics, immunity, and the microbiome—converges (13). Current studies increasingly suggest that these signaling perturbations interact through immune–vascular–metabolic pathways to disrupt cell-to-cell communication, feedback regulation, and structural stability at the maternal–fetal interface, thereby potentially impairing implantation or placental perfusion (43). To facilitate systematic analysis of pathological network reconstruction at the maternal–fetal interface in RPL, this section outlines a cross-hierarchical dysregulation framework based on three dimensions: disordered cell–cell interactions, aberrant signaling pathway cascades, and failure of microenvironmental remodeling.

### 4.1 Cell interaction disorders

DSCs and uNK cells jointly contribute to an immune-responsive environment during the implantation window through signaling pathways involving TGF-β–Smad and Galectin-9. This signaling context may promote the phenotypic transition of uNK cells from a more cytotoxic state to a trophoblast-supportive state, thereby permitting normal embryo invasion and implantation (44). In patients with RPL, interleukin-15 (IL-15) levels have been reported to be elevated, which may contribute to sustained activation of uNK cytotoxic programs (45). Concurrently, phosphorylation of Smad2 downstream of TGF-β in DSCs may be impaired, contributing to dysfunction of the local “immune tolerance axis” (46). Furthermore, abnormal uNK–trophoblast interactions have been associated with reduced Galectin-9 expression and imbalanced killer immunoglobulin-like receptor (KIR)–human leukocyte antigen-C (HLA-C) pairing, which may impair trophoblast invasiveness and endothelial remodeling, ultimately contributing to defective spiral artery remodeling and placental perfusion disorders (47, 48).

**Figure 2:**
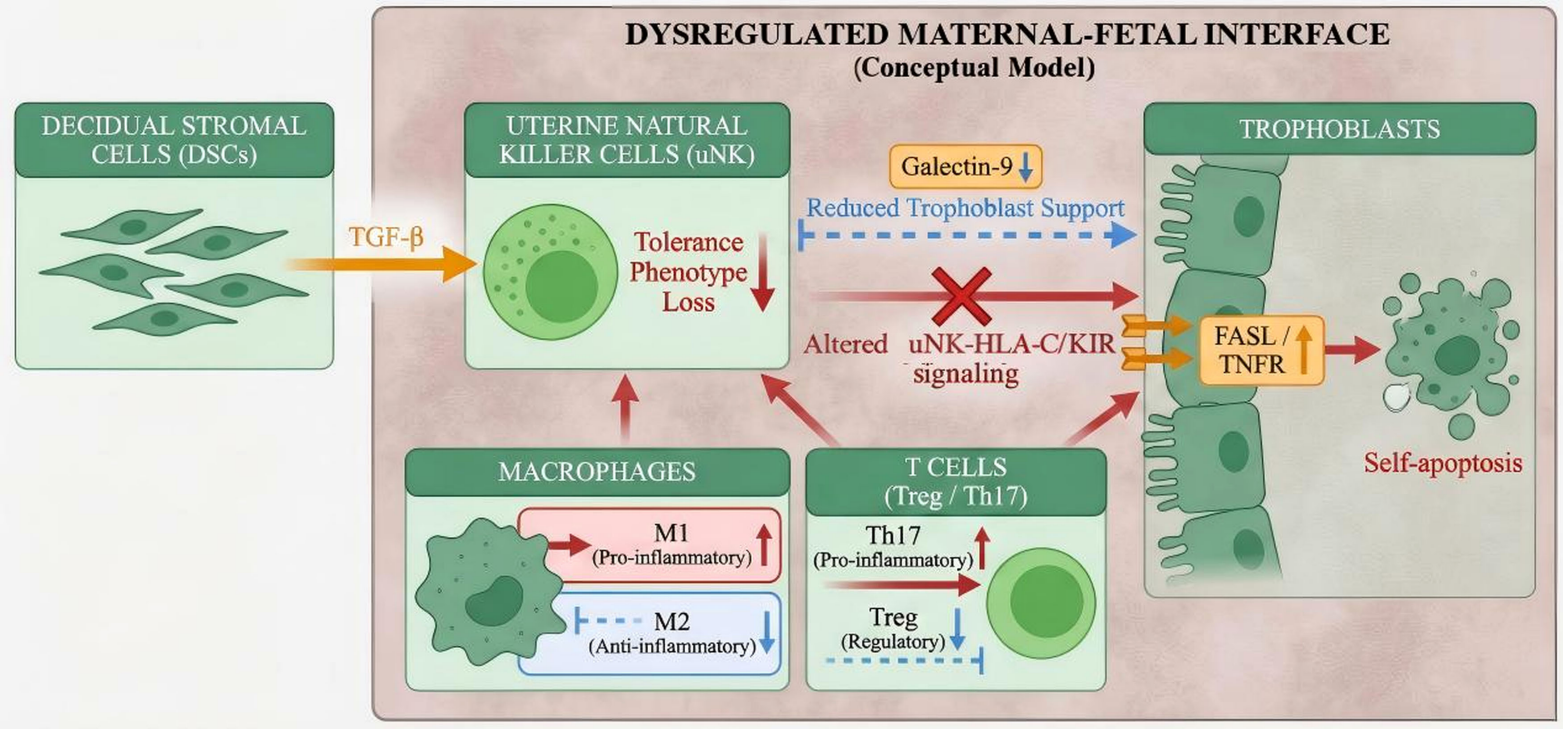
Disrupted cellular interaction network at the maternal–fetal interface in recurrent pregnancy loss (RPL). This schematic illustrates representative alterations in cell–cell communication associated with immune tolerance failure at the maternal–fetal interface. Under pathological conditions, impaired decidual stromal cell (DSC)-derived transforming growth factor-β (TGF-β) signaling may contribute to loss of the tolerogenic uterine natural killer (uNK) cell phenotype. Reduced Galectin-9 signaling and abnormal uNK–human leukocyte antigen-C (HLA-C)/killer immunoglobulin-like receptor (KIR) interactions may weaken trophoblast support. In parallel, macrophage polarization may shift toward a pro-inflammatory M1-like state, and the balance between regulatory T cells (Treg) and T helper 17 (Th17) cells may become disrupted. These alterations are associated with impaired trophoblast support and increased susceptibility to trophoblast stress and apoptosis. This figure is intended as a conceptual summary of reported cellular interaction abnormalities rather than a definitive mechanistic model. DSCs, decidual stromal cells; FASL, Fas ligand; HLA-C, human leukocyte antigen-C; KIR, killer cell immunoglobulin-like receptor; TGF-β, transforming growth factor-β; Th17, T helper 17 cells; TNFRs, tumor necrosis factor receptors; Treg, regulatory T cells; uNK, uterine natural killer cell.

Macrophages may also shift from an M2-dominant phenotype toward an M1-polarized state, accompanied by upregulation of pro-inflammatory markers such as cluster of differentiation 86 (CD86) and TNF-α, which may disrupt local anti-inflammatory balance and contribute to tissue injury and early pregnancy failure (49). Treg numbers may decrease and FOXP3 expression may be downregulated, permitting relative expansion of Th17-associated inflammatory responses and resulting in Treg/Th17 imbalance. A reduced Treg/Th17 ratio has been proposed as a high-risk immune phenotype in RPL (50). Notably, trophoblast cells may respond to maternal immune dysregulation by upregulating Fas ligand (FASL) and tumor necrosis factor receptors (TNFRs), thereby triggering apoptotic signaling and promoting trophoblast dysfunction (51). These cross-cell and cross-axis signaling abnormalities may collectively represent a central mechanism underlying disrupted cell interactions at the maternal-fetal interface in RPL (**Figure 2**).

However, most evidence for these interaction circuits is derived from small cohort studies, tissue-level observations, or preclinical models, and further validation is required to determine their reproducibility across biologically distinct RPL subtypes.

### 4.2 Abnormal signal pathway cascades

In RPL pathophysiology, abnormal cascades of inflammatory and metabolic signaling pathways are increasingly implicated in maternal–fetal interface dysfunction. The interleukin-17 (IL-17) axis has been reported to be activated in a subset of patients with RPL. Engagement of interleukin-17A (IL-17A) with the interleukin-17 receptor A/C (IL-17RA/RC) complex may contribute to activation of the transforming growth factor-β–activated kinase 1 (TAK1)–IκB kinase (IKK)–nuclear factor κ-light-chain-enhancer of activated B cells (NF-κB) signaling axis, thereby amplifying pro-inflammatory mediators—including IL-6, TNF-α, and interleukin-1β (IL-1β)—and promoting a self-reinforcing inflammatory cascade (52). NF-κB may also feedback to enhance IL-17A expression, thereby establishing a positive feedback loop that amplifies inflammatory signaling (52). TNF-α at the maternal–fetal interface may activate the Fas-associated protein with death domain (FADD)–caspase-8 (CASP8)–caspase-3 (CASP3) cascade within the extrinsic apoptosis pathway, thereby promoting programmed cell death in trophoblast and placental cells (53, 54), and may also exert anti-angiogenic effects (55). Clinical and pathological studies further suggest that placental microvessel rarefaction and impaired perfusion are associated with adverse pregnancy outcomes, including fetal growth restriction and preeclampsia (56).

Beyond inflammation, metabolic-immune dysregulation has also emerged as an important feature of RPL. Elevated homocysteine may promote the accumulation of reactive oxygen species (ROS) in trophoblasts and endothelial cells, suppress the superoxide dismutase (SOD)/glutathione peroxidase (GPx) antioxidant system, and activate oxidative stress pathways—including mitogen-activated protein kinase (MAPK) and the inositol-requiring enzyme 1α (IRE1α)–X-box-binding protein 1 (XBP1) axis—thereby increasing the Bcl-2-associated X protein (Bax)/B-cell lymphoma 2 (Bcl-2) ratio and contributing to mitochondria-dependent apoptosis (57, 58). Lipid metabolic disturbances may induce ceramide accumulation, which has been implicated in uNK activation and increased interferon-γ (IFN-γ) secretion, thereby promoting a T helper 1 (Th1)–skewed immune profile, attenuating Treg-mediated suppression, and amplifying inflammatory signaling (59).

**Figure 3:**
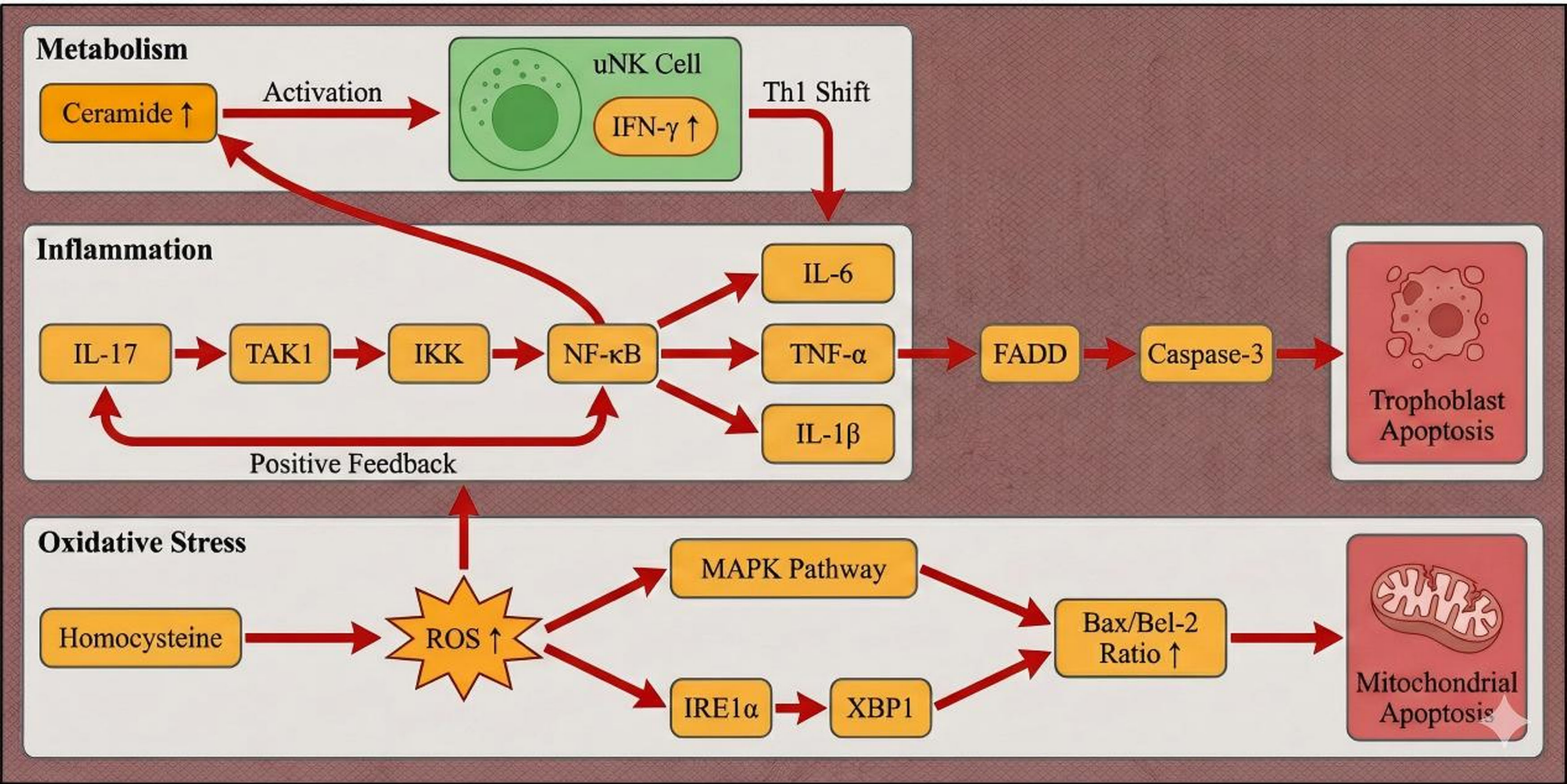
Representative inflammatory, metabolic, and oxidative stress signaling cascades implicated in recurrent pregnancy loss (RPL). This schematic summarizes several interconnected signaling modules frequently discussed in the context of RPL. The inflammatory axis includes interleukin-17 (IL-17)-associated activation of the TAK1–IKK–NF-κB pathway and downstream induction of interleukin-6 (IL-6), tumor necrosis factor-α (TNF-α), and interleukin-1β (IL-1β), which may contribute to trophoblast stress and apoptosis. The metabolic axis highlights ceramide accumulation and uterine natural killer (uNK) cell activation with interferon-γ (IFN-γ) production and T helper 1 (Th1)-skewed immune signaling. The oxidative stress axis depicts homocysteine-associated reactive oxygen species (ROS) accumulation, activation of MAPK and IRE1α–XBP1 pathways, and mitochondrial apoptotic signaling. This figure summarizes putative signaling interactions derived primarily from preclinical and mechanistic studies and is intended to illustrate pathway convergence rather than definitive causal hierarchy. uNK, uterine natural killer cell; IFN-γ, interferon gamma; Th1, T helper 1 cell; IL-17, interleukin-17; NF-κB, nuclear factor κ-light-chain-enhancer of activated B cells; TAK1, transforming growth factor-β–activated kinase 1; IKK, IκB kinase; IL-6, interleukin-6; TNF-α, tumor necrosis factor alpha; IL-1β, interleukin-1 beta; FADD, Fas-associated protein with death domain; ROS, reactive oxygen species; MAPK, mitogen-activated protein kinase; IRE1α, inositol-requiring enzyme 1 alpha; XBP1, X-box-binding protein 1; Bax, Bcl-2–associated X protein; Bcl-2, B-cell lymphoma 2.

In summary, immunity, metabolism, and apoptosis pathways in RPL appear to be highly interconnected, forming a signaling cascade from cellular perception to pathological output. Dysregulation across multiple biological axes may represent an important determinant of interface dysfunction (**Figure 3**).

Nevertheless, many of these pathway models are supported primarily by preclinical or associative evidence, and their causal relevance in human RPL requires further validation.

### 4.3 Failure of microenvironment remodeling

When assessed using spatial omics and hemodynamic profiling, decidual tissues from patients with RPL have been reported to contain regions with reduced Treg cell abundance and lower IL-10 expression, alongside nearby foci enriched in inflammatory mediators such as interleukin-8 (CXCL8) and IFN-γ, indicating marked local immune heterogeneity (60). Within the VEGFR2–phosphoinositide 3-kinase (PI3K)–endothelial nitric oxide synthase (eNOS) signaling axis, impaired signaling activity has been reported to contribute to defective endothelial migration, branching, and vascular adaptation during early pregnancy. Disrupted spiral artery remodeling and abnormal uteroplacental perfusion may promote intermittent ischemia–reperfusion injury, thereby enhancing the release of damage-associated molecular patterns (DAMPs) and ROS. These signals may further activate innate immune pathways, including TLR4-mediated signaling and NLRP3 inflammasome activation, ultimately amplifying inflammatory responses at the maternal–fetal interface (61). The inflammatory–oxidative amplification loop may further destabilize the decidual microenvironment (62).

Based on the above-mentioned multi-omics abnormalities and disrupted cellular interactions, RPL may be conceived as a disorder involving interrelated regulatory axes of endometrial receptivity, trophoblast invasion, vascular remodeling, and immune tolerance. Perturbation in any one of these axes, such as reduced Treg-mediated suppression or attenuated VEGF signaling, may propagate through interconnected pathways and contribute to a progressively pathological state. Four major defect points in interactions are found:

(1) Dysregulation of the uNK–DSC axis: reduced TGF-β signaling may impair the transition of uNK cells toward an immunoreceptive phenotype, thereby reducing implantation competence (44);
(2) Persistent IL-17/TNF-α network activation: NF-κB may amplify proinflammatory signaling, suppress angiogenesis-related pathways, and contribute to activation of FADD–caspase cascades (63);
(3) Loss of Treg stability: FOXP3 downregulation with relative Th1/Th17 polarization may disrupt local immune tolerance and thereby compromise endometrial receptivity (64);
(4) Ceramide-induced oxidative stress: accumulated ceramides may increase ROS levels and active MAPK and IRE1α–XBP1 signaling, thereby contributing to cell death and disrupted decidual architecture (65).

**Figure 4:**
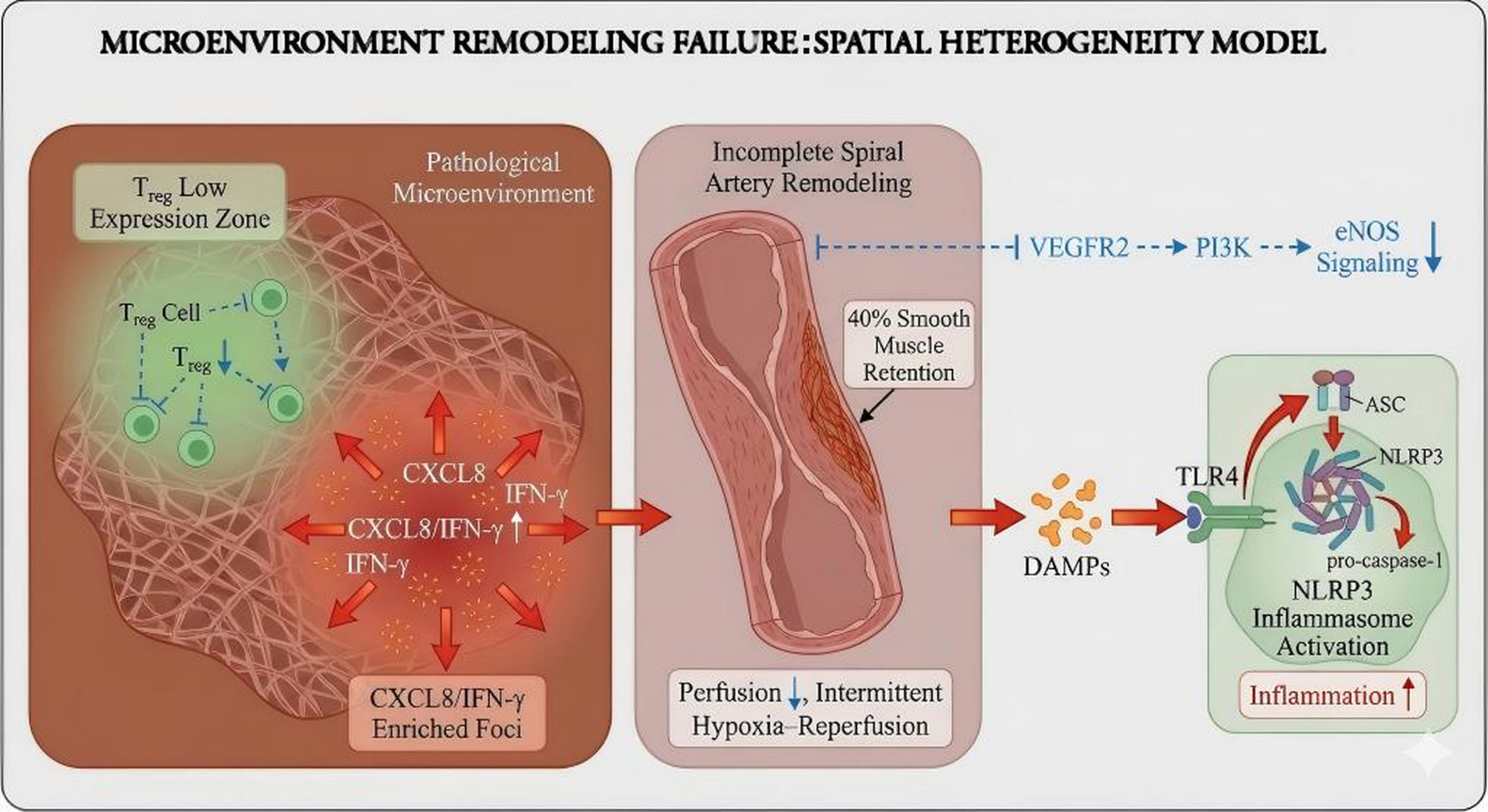
Conceptual model of microenvironment remodeling failure and spatial immune heterogeneity at the maternal–fetal interface in recurrent pregnancy loss (RPL). This schematic illustrates how focal immune heterogeneity, vascular maladaptation, and innate immune activation may interact within the decidual microenvironment. Regions with reduced regulatory T-cell (Treg) activity and increased CXCL8/interferon-γ (IFN-γ)-enriched inflammatory signaling may coexist with impaired spiral artery remodeling and reduced VEGFR2–PI3K–eNOS pathway activity. These abnormalities may promote hypoperfusion and intermittent ischemia–reperfusion stress, leading to the release of damage-associated molecular patterns (DAMPs) and subsequent activation of innate immune pathways such as Toll-like receptor 4 (TLR4) and the NLRP3 inflammasome. This figure is intended to illustrate a spatially organized pathological framework rather than a definitive linear mechanism. RPL, recurrent pregnancy loss; Treg, regulatory T cell;CXCL8, C-X-C motif chemokine ligand 8 (interleukin-8);IFN-γ, interferon gamma;VEGFR2, vascular endothelial growth factor receptor 2;PI3K, phosphoinositide 3-kinase;eNOS, endothelial nitric oxide synthase; PI, pulsatility index; DAMPs, damage-associated molecular patterns;TLR4, Toll-like receptor 4;NLRP3, NOD-like receptor family pyrin domain–containing 3;ASC, apoptosis-associated speck-like protein containing a CARD.

Future studies should work toward developing multiscale dynamic models that integrate signaling networks, cell–cell interactions, and tissue remodeling. Combining spatial multi-omics with AI-based network inference may help reconstruct hierarchical interaction maps linking molecular perturbations to tissue homeostasis, thereby informing future target identification and coordinated multi-axis therapeutic exploration (**Figure 4**). However, these microenvironmental models remain inferential in many settings, and further integration of spatial, functional, and longitudinal human data will be necessary to determine their robustness across different RPL phenotypes.

## 5 Progress in targeted intervention strategies and translational research

With advances in multi-omics technologies for analyzing the pathogenic mechanisms of RPL, key pathological nodes have gradually expanded from genetic susceptibility and metabolic abnormalities to cell–cell interaction disorders and signaling cascade dysregulation, thereby creating new opportunities to move beyond purely empirical intervention toward more mechanism-informed management. Currently, emerging multi-target regulatory approaches addressing abnormal signals at the maternal–fetal interface span multiple dimensions, including modulation of molecular pathways, reconstruction of the immune microenvironment, correction of metabolic dysregulation, exosome-mediated delivery systems, and AI-assisted prediction frameworks.

This section summarizes representative intervention pathways and their translational potential from four perspectives, and outlines a conceptual framework linking mechanism, strategy, and potential application to support future development of individualized diagnostic and therapeutic models for RPL.

### 5.1 Epigenetic regulation and immune remodeling

With increasing understanding of maternal–fetal immune imbalance, epigenetic regulation and interventions targeting the Treg/Th17 axis have emerged as promising strategies in RPL research. Current evidence suggests that Treg/Th17 imbalance is among the most frequently reported immune abnormalities in RPL (66). Current intervention concepts in this area generally aim to enhance Treg function or inhibit Th17 expansion.

Low-dose interleukin-2(IL-2), vitamin D3, and tranilast have been reported to increase Treg abundance and upregulate FOXP3 expression in preclinical or early translational settings (67, 68). Induction of regulatory dendritic cells (DCregs) to promote Treg homing and activation has shown beneficial pregnancy-maintenance effects in RPL mouse models (69). Conversely, neutralizing IL-17 with agents such as secukinumab, or blocking retinoic acid receptor-related orphan receptor-γt (RORγt) with digoxin, has been reported to prolong gestational duration and partially restore the immune microenvironment in animal models (70, 71). Some recent studies have reported that IL-6/IL-17 bispecific antibodies may show greater efficacy than single-target strategies in suppressing inflammation and promoting tolerance development, although current evidence remains preliminary (72). At the epigenetic level, aberrant patterns—such as hypermethylation of the FOXP3 promoter or reduced histone H3 lysine 27 acetylation (H3K27ac)—may be pharmacologically modulated by demethylating agents (e.g., 5-azacytidine [5-Aza]) or histone deacetylase (HDAC) inhibitors (e.g., suberoylanilide hydroxamic acid [SAHA]). These interventions may indirectly enhance Treg-associated regulation and help restore a microenvironment more favorable to immune tolerance (73). High-precision epigenetic tools, such as clustered regularly interspaced short palindromic repeats–dead CRISPR-associated protein 9 (CRISPR–dCas9) platforms, have been explored to target inhibitory marks on the FOXP3 promoter, indicating the conceptual feasibility of targeted epigenetic manipulation in RPL, although this approach remains preclinical (74). Future work should focus on evaluating whether multimodal immunotherapies combined with epigenome editing can safely and effectively support reconstruction of immune regulation at the maternal–fetal interface.

Overall, most approaches in this area remain at the preclinical or early proof-of-concept stage, and their translational applicability in human RPL requires further validation.

### 5.2 Targeting oxidative stress and metabolic abnormalities

In RPL, oxidative stress and abnormal lipid metabolism appear to interact as an important network contributing to cellular dysfunction. Elevated ROS may induce apoptosis of trophoblast and decidual cells via the MAPK–caspase pathway and promote sustained secretion of inflammatory cytokines, including TNF-α and IL-1β. Together, these processes may form a positive feedback loop of inflammation and local tissues damage (75). Antioxidant therapy has emerged as a potential approach for mitigating oxidative stress–associated damage. Compounds such as N-acetylcysteine (NAC), coenzyme Q10 (CoQ10), and vitamin E have been reported to reduce malondialdehyde (MDA) levels and enhance SOD activity in decidual tissues, thereby attenuating decidual apoptosis in preclinical or limited clinical settings (76). Mitochondria-targeted repair compounds, including SS-31 and MitoQ, have been reported to restore mitochondrial membrane potential, increase ATP synthesis, and enhance Bcl-2 expression in trophoblasts in experimental models. These findings provide a rationale for further exploration of mechanism-based therapies in oxidative stress–associated RPL (77, 78). Dimethyl fumarate and other molecules that activate nuclear factor erythroid 2–related factor 2 (Nrf2) have been shown to enhance decidual antioxidant defense in animal models (79). In settings of disordered lipid metabolism, decidual tissues in RPL have been reported to accumulate ceramides. This lipid imbalance may promote uNK-associated IFN-γ production and shift the Th1/T helper 2 (Th2) equilibrium toward a more pro-inflammatory state (80, 81). Pharmacologic inhibition of ceramide synthesis (e.g., myriocin, a serine palmitoyltransferase inhibitor) and modulation of lipid droplet metabolism (e.g., T863, a DGAT1 inhibitor) have been reported to attenuate lipotoxicity-induced immune activation in experimental systems (82). In addition, agonists of peroxisome proliferator-activated receptor γ (PPARγ) such as rosiglitazone, may reduce lipid droplet overload and suppress local inflammation. By impeding NF-κB-dependent transcription of inflammatory mediators such as IL-6 and monocyte chemoattractant protein-1(MCP-1), these agents may contribute to re-establishing a more tolerogenic immune microenvironment (83). Ferroptosis, a form of iron-dependent, lipid peroxidation-driven programmed cell death, has recently emerged as a candidate mechanism in RPL. Decidual tissues from affected pregnancies have been reported to show decreased GPX4 activity and excess accumulation of Fe^2+^, which may contribute to lipid peroxidation and mitochondrial injury. Therapeutic agents such as deferoxamine (DFO), an iron chelator, and ferrostatin-1 (Fer-1), a ferroptosis inhibitor, have been shown in preclinical models to reduce ferroptosis, preserve cellular function at the maternal–fetal interface, and prolong pregnancy duration (84). Collectively, disturbances in oxidative stress, lipid metabolism, and ferroptosis appear to form a metabolic disruption loop that may aggravate mitochondrial dysfunction, inflammatory amplification, and progression of cellular injury in RPL. Future work should focus on evaluating cross-pathway metabolic modulators and phenotype-matched approaches to support the development of more individualized, mechanism-informed therapeutic strategies.

However, many of these interventions remain supported primarily by animal or exploratory studies, and their safety, timing, and applicability in human RPL require further study.

### 5.3 Exosomes and cytokine delivery systems

Trophoblast-derived exosomes function as important intercellular communicators at the maternal–fetal interface. They also carry a repertoire of immunoregulatory factors such as TGF-β and regulatory microRNAs, including microRNA-146a (miR-146a) and microRNA-155 (miR-155), that may help establish and maintain an immune-tolerant environment in early pregnancy (85, 86). Studies have reported that the level of miR-146a in serum and decidual exosomes is reduced in patients with RPL, suggesting possible involvement in maternal immune activation and interface disruption (86–88). Exosome engineering technologies based on mesenchymal stromal cells(MSCs) have been developed to enhance their regulatory capacity. Functional RNAs such as miR-146a loaded into engineered MSC-derived exosomes can be delivered to maternal immune cells, where they have been shown in animal models to promote immune restoration and pregnancy maintenance (88–90). Cytokine delivery platforms based on liposomes, chitosan, and polypeptide carriers may enable localized release of immunoregulatory molecules such as interleukin-10 (IL-10) and programmed death-ligand 1 (PD-L1) within decidual models, thereby enhancing targeted immune modulation in experimental systems (91–93). Compared with conventional small molecules, these systems may offer advantages in local immunomodulatory efficacy, tissue compatibility, and delivery specificity, although direct comparative evidence remains limited. The Arg-Gly-Asp (RGD) peptide can be incorporated to enhance binding to integrin-rich regions at the trophoblast-decidual interface, potentially improving local accumulation of the delivery system at the maternal-fetal interface (94–96). The surface of these engineered exosomes may also be modified to improve tissue penetration and targeted delivery. These approaches may provide a platform for future individualized immune intervention design. Additionally, exosomes are also being explored as biomarkers for monitoring therapeutic response in RPL (93, 97). High-throughput profiling of serum exosomal microRNAs may allow dynamic and noninvasive monitoring of treatment response, immune state, and maternal-fetal interface homeostasis (98, 99). Such methods may provide a feasible route for linking diagnosis, therapy, and monitoring within a more coherent translational framework.

At present, however, most exosome-based and cytokine-delivery approaches remain preclinical, and issues related to manufacturing consistency, biodistribution, and long-term safety must be addressed before clinical translation.

### 5.4 Precise predictive models and the timing of interventions

Recent advances in multi-omics have begun to reshape the conceptual framework of RPL management from empirical approaches toward more structured strategies involving prediction, stratification, and follow-up. Multi-omic scoring models based on serum microRNAs, metabolite markers, and immune cell populations, combined with machine-learning techniques such as XGBoost or LASSO logistic regression, have shown promise for improving early risk detection. In some cohorts, these models have achieved an area under the curve exceeding 0.92,outperforming single-marker approaches (100–103). Further multimodal early warning systems integrating ultrasound perfusion parameters, miscarriage history, and omics-based features may help classify RPL into biologically meaningful subtypes, such as inflammation-dominant, metabolism-dominant, or vascular-perfusion–dominant patterns (17, 104) (105). Graph-based neural network frameworks and metabolic–immune–structure integrated atlases may provide a means to monitor individual risk trajectories over time, thereby helping to inform targeted intervention planning (106, 107). Time-series neural networks (TSNNs) and dynamic Bayesian networks (DBNs), by modeling cyclic immune dynamics, may enable prediction of inflection points such as Treg decline or reduced vascular perfusion, thereby helping identify potential windows for timely intervention (108–110).

**Figure 5:**
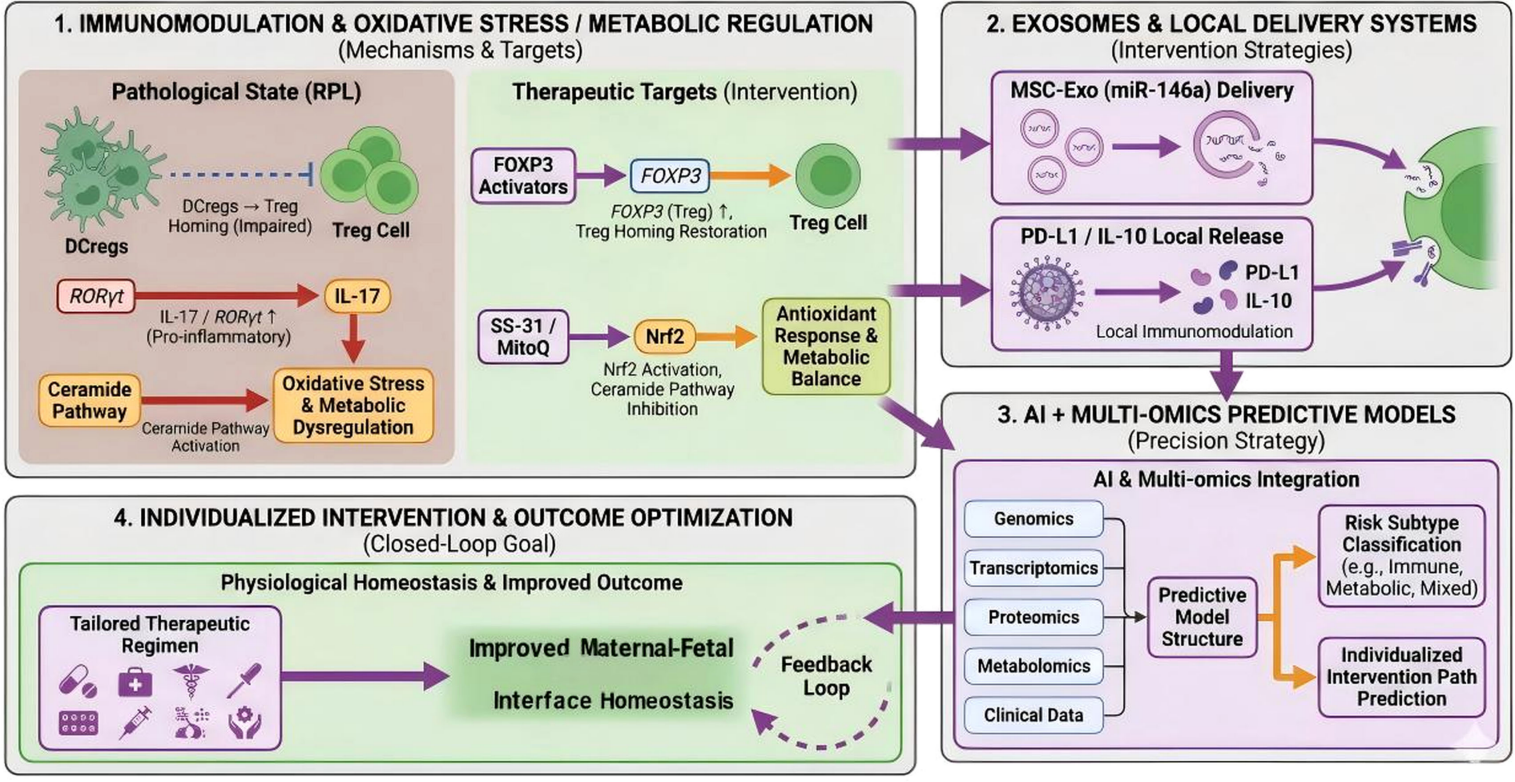
Conceptual framework for targeted intervention and translational strategies in recurrent pregnancy loss (RPL). This schematic summarizes four interconnected domains of emerging intervention strategies: (1) immunomodulation and oxidative stress/metabolic regulation, including Treg-supportive and antioxidant approaches; (2) exosome-based and local delivery systems for targeted immune modulation; (3) artificial intelligence (AI)-assisted and multi-omics-based predictive models for subtype classification and individualized risk assessment; and (4) individualized intervention and outcome optimization through feedback-informed management. The figure is intended to illustrate a translational roadmap linking mechanistic insights to precision reproductive medicine. Most strategies shown remain at preclinical or early translational stages and should not be interpreted as established clinical standards of care. RPL, recurrent pregnancy loss; DCreg, regulatory dendritic cell; Treg, regulatory T cell; RORγt, retinoic acid–related orphan receptor gamma t;IL-17, interleukin-17;FOXP3, forkhead box P3;Nrf2, nuclear factor erythroid 2–related factor 2;MSC-Exo, mesenchymal stromal cell–derived exosomes;miR-146a, microRNA-146a;PD-L1, programmed death-ligand 1;IL-10, interleukin-10;AI, artificial intelligence.

In other words, as these analytical tools become more refined, AI-augmented platforms may become better able to track an individual’s response to treatment and iteratively refine predictive outputs based on feedback. Accordingly, intervention concepts are gradually shifting from broadly restorative approaches toward more mechanism-based, phenotype-matched, and time-sensitive strategies. As advances continue in multi-omics diagnostics, spatial tissue mapping, and targeted delivery systems, it may become increasingly feasible to monitor and modulate pathological processes at the maternal–fetal interface, thereby supporting more individualized care in the future (**Figure 5**).

Nonetheless, most predictive and timing-oriented models remain exploratory, and their prospective performance, generalizability, and impact on clinical outcomes require formal validation.

## 6 Future prospects and research challenges

Although recent advances in multi-omics profiling, spatial transcriptomics, AI, and nanomaterial-based delivery systems have improved our understanding of RPL and opened new avenues for mechanistic investigation and translational exploration, substantial challenges remain. RPL should not be regarded as a single-source entity because of its pronounced heterogeneity across phenotypes, biological layers, and time, which hinders comprehensive mechanistic understanding. Current limitations in mechanistic resolution, variability in diagnostic and predictive performance across platforms, and the absence of well-defined criteria for patient-specific intervention selection continue to impede clinical translation. These challenges must be addressed before technological advances can be translated into mechanism-based and individualized management strategies.

### 6.1 Transition from phenotypic classification to mechanism-informed models

Current classification systems for RPL mainly rely on broad phenotypic features such as immune-cell composition and secreted products, hormones and their metabolites, inflammatory mediators, and other molecular signals associated with RPL. While several clinical subtypes have been proposed, such as “high immune-response,” “low progesterone,” and “chromosomal abnormality” types, these groupings are still largely based on relatively static observable features. Therefore, most current classification systems remain descriptive and provide limited guidance for targeted intervention selection.

Accordingly, a shift toward more mechanism-informed classification models is needed. Multi-omics technologies provide a potential means to integrate information across cells, molecules, and spatial contexts into models that connect etiology, mechanisms, and therapeutic responsiveness. Spatial transcriptomics, single-cell sequencing, and time-series models may capture dynamic patterns of cell–cell communication and signaling across different stages of early pregnancy, thereby helping distinguish causal from consequential processes.

For example, coupling metrics such as temporal changes in the Treg/Th17 ratio, activity of the uNK–DSC axis, signaling strength in the VEGF–eNOS pathway, and indicators of mitochondrial metabolic performance could potentially be used to construct a mechanism-based “RPL interaction map”. Such a system could help define functional subtypes based on pathogenic processes rather than surface phenotype alone. This could support earlier risk prediction and more mechanism-oriented therapeutic exploration.

In addition, translational tools and intervention platforms suitable for such a framework need to be developed to bridge the gap from diagnostic results to etiological interpretation, and from etiological interpretation to treatment planning, thereby providing a more actionable basis for mechanism-oriented and individualized management of RPL.

### 6.2 Construction of a dynamically traceable biomarker system

Currently, most RPL-related biomarkers are derived from static sampling at specific pregnancy time points, making it difficult to capture the dynamic immune, metabolic, and hemodynamic processes at the maternal-fetal interface during early pregnancy. Future research should focus on establishing a longitudinal sampling framework encompassing multiple time points, multiple pathways, and multiple body-fluid sources together with development of dynamic predictive biomarkers using liquid-biopsy platforms such as exosomal miRNA, cell-free DNA, and metabolomic signals. Preliminary studies suggest that dynamic changes in exosomal miRNA may reflect shifts in pregnancy immune status, highlighting their potential utility in RPL risk prediction. Dynamic monitoring of the microenvironment and intervention responses may provide important support for individualized early warning, identification of optimal intervention windows, and adaptive decision-making in RPL.

### 6.3 Transformation bottlenecks in individualized intervention

Although various intervention strategies for RPL have shown positive efficacy in animal models, their clinical translation faces numerous obstacles. A major limiting factor is the high heterogeneity of pathological features at the maternal-fetal interface, which complicates the formulation of broadly applicable intervention plans. Additionally, the absence of large-scale, multicenter confirmatory clinical trials limits both the evidence level and the feasibility of widespread implementation. Individual differences in pregnancy timing, physiological status, and drug response further complicate standardized decisions regarding intervention timing and dosage. The large-scale clinical application of core technologies, such as targeted delivery systems, remains at an early stage. To shift interventions from experience-oriented to data-informed approaches, a multidimensional intervention-response database encompassing patient characteristics, treatment pathways, and pregnancy outcomes should be established, alongside AI-assisted modeling to support an individualized prediction–response–feedback framework RPL intervention.

### 6.4 System simulation path by integrating digital twin and AI modeling

RPL is a highly heterogeneous disorder at the network level and involves multiple interacting biological systems. There is increasing interest in developing computational models to capture pathological processes across multiple tissue levels and time points during pregnancy. Emerging applications of digital twin technology in reproductive medicine are attempting to create individualized, data-based virtual pregnancy models that approximate the real-time state of the maternal–fetal interface. Such models may enable longitudinal monitoring of pathological changes and prediction of individual responses to specific interventions.

Integration of AI, particularly through combined learning with multi-omics, spatial omics, histopathological imaging, and other multimodal data, may further improve the accuracy and generalizability of system modeling. This technological approach may gradually shift RPL management from traditional *post-hoc* identification toward earlier prediction and more mechanism-informed monitoring, thereby providing a conceptual foundation for precision reproductive medicine. Future development of a digital simulation platform integrating individual data, mechanism modeling, and intervention evaluation may serve as an important tool for individualized management of RPL.

### 6.5 Potential of uterine microecological regulation in RPL intervention

In recent years, research has increasingly challenged the traditional view that the uterus is a sterile cavity, suggesting the existence of specific microbial communities in the uterine environment that may influence embryo receptivity, local immune homeostasis, and signal transduction. Multiple studies based on 16S rRNA sequencing have reported reduced *Lactobacillus* abundance in patients with RPL, accompanied by overrepresentation of *Gardnerella* and *Atopobium* genera, suggesting an association between uterine microbial dysregulation and RPL (111–116).

Mechanistically, microecological imbalance may weaken immune receptivity during the implantation window by activating Toll-like receptor signaling, inducing IL-1β-mediated local inflammatory responses, or interfering with the homeostatic function of uterine NK cells. Based on these insights, novel microecological regulation methods—such as intrauterine probiotic implantation and delivery of intrauterine antimicrobial peptides—have been proposed as potential next-generation individualized intervention strategies with more localized effects. Future research should further explore the dynamic succession patterns of uterine microbial communities, integrate spatial immunomics with hormone receptor expression profiles, and construct a three-dimensional regulatory framework linking microecology, immunity, and endocrinology. Establishing a microecology-based regulatory model integrating immune and endocrine signals may help identify biomarkers, support early pregnancy monitoring, and guide future individualized microecological intervention strategies.

### 6.6 Construction of an interdisciplinary, multi-layered intervention framework

Conventional drugs or immunomodulators developed for RPL treatment often exhibit limited targeting specificity, uncertain efficacy, and potential risks of off-target systemic toxicity when applied to the highly complex and dynamic maternal–fetal interface. Future therapeutic progress is likely to rely on stronger interdisciplinary collaboration, particularly across biomaterials science, tissue engineering, stem cell therapy, and systems biology, in order to address the heterogeneous nature of disease mechanisms.

Emerging three-dimensional (3D) uterine organoid platforms may function as experimental systems for modeling epithelial–immune–vascular interactions and testing responses in a controlled setting. These 3D uterine organoids may allow observation of specific mechanistic perturbations and evaluation of individualized responses to potential treatments. Meanwhile, engineered mesenchymal stem cell exosome delivery systems and intelligent controlled-release nanocarriers that respond to inflammatory or metabolic changes have demonstrated promising targeting, biocompatibility, and intervention efficiency in animal models. In the future, the RPL intervention paradigm may shift from single-pathway repair toward more comprehensive strategies centered on system reprogramming, multi-target coordination, and dynamic feedback regulation, thereby providing stronger technical support and mechanistic foundations for individualized RPL management.

### 6.7 Strengthening global data integration and standardization in RPL research

Significant heterogeneity exists in key aspects of RPL research, including disease definition, clinical classification, diagnostic procedures, and sample collection. These disparities substantially limit data integration and model generalizability across research centers. Specifically, there is a lack of unified technical standards and data-sharing agreements regarding multi-omics platform compatibility, spatial omics data structure standardization, and pregnancy timing modeling. Additional challenges, such as differences in ethical review standards, non-standardized sample annotations, and incomplete clinical follow-up information, further compromise reproducibility and the generalizability of AI models. Future RPL research should leverage global multicenter cohorts (e.g., the Human Cell Atlas and the IMPRINT network) to establish comprehensive standards encompassing sampling time windows, storage conditions, sequencing platforms, data formats, metadata annotation, and analysis interfaces. A data collaboration system adhering to FAIR principles (Findable, Accessible, Interoperable, Reusable) should be implemented, alongside an updated ethical review framework that balances privacy protection, traceability, interpretability, and responsible sharing of reproductive omics data. Ultimately, internationally recognized collaborative networks for RPL research should be established.

### 6.8 Ethical and data-compliance challenges in multi-omics research on RPL

The application of multi-omics in RPL research has introduced a range of complex ethical and regulatory challenges, particularly in relation to the collection and use of sensitive reproductive information. The acquisition of embryonic tissues, decidual samples, and pregnancy-related liquid biopsies requires strict ethical oversight, with careful attention to informed consent, sample ownership, and scientific justification for specimen collection. As more studies focus on metabolic, reproductive, and immune communication systems, cross-institutional sharing of sensitive health information under regulated consent frameworks is becoming increasingly important.

Longitudinal studies incorporating clinical data, pregnancy course, and treatment responses raise important questions regarding anonymization, de-identification, and authorization for long-term follow-up. Although standardized datasets for AI-based predictive models are increasing, inconsistencies in sequencing platforms and in sample and metadata labeling practices across centers continue to reduce the reproducibility and usability of these models.

To address these problems, international collaborative projects such as the Human Cell Atlas and the Global Alliance for Genomics and Health (GA4GH) provide useful reference frameworks for standardizing sampling frequency, informed consent processes, data formats, and governance structures. The development of interoperable regulatory frameworks will be essential to enable responsible multi-omics data integration and eventual translation of mechanistic discoveries.

### 6.9 Evidence-based mapping of representative interventions

To clarify the current translational landscape, **Table 1** summarizes representative intervention strategies according to their primary mechanism, evidence level, delivery route, and potentially relevant RPL phenotype. Importantly, most of these approaches remain preclinical or exploratory and should be interpreted as hypothesis-generating rather than established standards of care.

**Table 1:** Evidence-based mapping of representative intervention strategies for recurrent pregnancy loss (RPL).

Intervention type	Primary target/mechanism of action	Evidence level	Delivery route	Potentially applicable RPL subtype	Key references
Low-dose IL-2/Vitamin D3 (Treg-supportive immunomodulation)	Expansion of FOXP3+ regulatory T-cell (Treg); suppression of Th1/Th17-associated inflammation; partial restoration of immune tolerance	Preclinical with limited early clinical observations	Subcutaneous or intravenous low-dose IL-2; oral vitamin D3	Immune-dysregulated/Treg-low/Th17-skewed phenotype)	(117)
Epigenetic modulation (5−AZA, SAHA)	FOXP3 promoter demethylation; chromatin remodeling; enhancement of Treg transcriptional program and immune tolerance-associated gene expression	Preclinical(animal and mechanistic studies)	Systemic administration; exploratory local/intrauterine delivery	Epigenetic–immune dysregulation phenotype	(118)
Antioxidants (N-acetylcysteine, MitoQ)	Reduction of reactive oxygen species (ROS); modulation of Bax/Bcl-2 ratio; mitochondrial protection and attenuation of oxidative stress–associated injury	Early clinical/exploratory evidence for N-acetylcysteine; preclinical evidence for MitoQ	Oral or intravenous administration	Oxidative-stress dominant/metabolic stress phenotype	(119)
Exosome-based therapy (miR-146a–engineered exosomes)	Suppression of IL-6/IL-17-associated inflammatory signaling; restoration of local immune homeostasis; targeted immune modulation	Preclinical (animal and mechanistic studies)	Exosomal carrier; exploratory local/intrauterine delivery	Inflammatory/immune-dominant delivery	(120)
Ferroptosis modulation (ferrostatin-1)	Restoration of glutathione peroxidase 4 (GPX4) activity; reduction of Fe^2+^-associated lipid peroxidation; protection against ferroptotic tissue injury	Exploratory/proof-of-concept preclinical evidence	Systemic treatment; exploratory nanoparticle-based delivery	Oxidative-stress/tissue injury–associated phenotype	(121)
Adoptive Treg transfer (cell therapy)	Direct replenishment of FOXP3+ Treg pool; restoration of maternal–fetal immune tolerance	Preclinical with limited early translational exploration	Intravenous infusion; exploratory intrauterine cell delivery	Immune-driven tolerance-defect phenotype	(122)
Tolerogenic dendritic cells (tDCs)	Upregulation of interleukin-10 (IL-10); suppression of Th17-associated inflammation; induction of Treg homing and activation	Preclinical (animal and mechanistic studies)	Local/intrauterine delivery; exploratory cell infusion	Immune-imbalance phenotype	(123)
Nrf2 activators (sulforaphane, bardoxolone)	Activation of the nuclear factor erythroid 2–related factor 2 (Nrf2)–antioxidant response element (ARE) pathway; enhancement of antioxidant and detoxification responses	Preclinical evidence	Oral or systemic administration	Oxidativestress–dominant phenotype	(124)
AMPK activators (metformin, AICAR)	Improvement of cellular energy metabolism; partial restoration of metabolic homeostasis and interface stress adaptation	Early Clinical/exploratory evidence for metformin; limited preclinical support for AICAR in this context	Oral administration	Metabolic/insulin-resistance– associated phenotype	(125)
Microbiome-based interventions (probiotics)	Restoration of gut–reproductive tract microbial balance; modulation of immune–microbial crosstalk and systemic immune tone	Early clinical/exploratory evidence	Oral administration	Inflammatory/microbiome-associated immune dysregulation phenotype	(27)

RPL, recurrent pregnancy loss; uNK, uterine natural killer; DSCs, decidual stromal.

cells; Treg, regulatory T cell; Th17, T helper 17 cell; TLR4, Toll-like receptor 4; MyD88, myeloid differentiation primary response 88; NF-κB, nuclear factor kappa B; MAPK, mitogen-activated protein kinase; NLRP3, NOD-like receptor family pyrin domain containing 3; ROS, reactive oxygen species; DAMPs, damage-associated molecular patterns.

## 7 Conclusion

Taken together, multi-omics and spatial immunology studies increasingly support the view that RPL can be understood as an immune-network disorder rather than merely a collection of isolated pathological events.

RPL is a biologically heterogeneous disorder characterized by converging abnormalities in genetics, epigenetic regulation, immunity, metabolism, vascular remodeling, and microecology at the maternal–fetal interface. With the increasing use of multi-omics technologies, the field is moving toward a more integrated understanding marked by a shift from phenotype-based description to mechanism-oriented interpretation. Genomic evidence, single-cell transcriptomic data, spatially resolved profiling, metabolomic data, and immunophenotypic analyses have begun to identify potentially reproducible molecular signatures and interaction networks, providing a more organized view of early pregnancy failure. These advances have also accelerated progress in biomarker discovery and risk-stratification model development, as well as in exploration of new therapeutic strategies, including immune modulation, metabolic correction, and exosome-based delivery concepts.

Nevertheless, several major challenges still restrict translation into clinical practice. Current studies are frequently based on relatively small and heterogeneous cohorts, with insufficient temporal sampling and sequencing or analytical pipelines that lack standardization. Differences in diagnostic concepts, together with variations in sample processing and annotation across research institutions, further reduce generalizability. In addition, many mechanistic insights remain far from clinically actionable treatment options, and very few intervention approaches have been evaluated in rigorous large-scale prospective trials. These limitations highlight the need for unified sampling frameworks, standardized multi-omics data structures, and ethically appropriate international data-sharing systems.

Future work should focus on three major goals. First, mechanistic subtyping should be strengthened by combining spatial and longitudinal omics with dynamic models of maternal–fetal interface signals, rather than interpreting downstream correlates as causal drivers. Second, predictive systems that integrate multi-omics features with machine learning and digital-twin simulations may help identify optimal intervention windows and support more individualized treatment decisions. Third, multilayered interaction maps involving uterine microecological structure and function, immune tolerance pathways, endocrine regulation, and vascular-metabolic dynamics should be developed to better conceptualize the mechanisms underlying RPL.

Ultimately, the goal is to translate mechanistic discoveries into clinically usable tools through multicenter collaboration, protocol standardization, and robust translational pipelines, thereby supporting earlier detection, more mechanism-informed intervention, and improved reproductive health for women affected by RPL. Such integration may gradually shift the clinical paradigm of RPL from reactive management toward more proactive and immune-informed care.
